# Cancer detection rates of the PI-RADSv2.1 assessment categories: systematic review and meta-analysis on lesion level and patient level

**DOI:** 10.1038/s41391-021-00417-1

**Published:** 2021-07-06

**Authors:** Benedict Oerther, Hannes Engel, Fabian Bamberg, August Sigle, Christian Gratzke, Matthias Benndorf

**Affiliations:** 1 Department of Radiology, Medical Center - University of Freiburg, Faculty of Medicine, University of Freiburg, Germany, Freiburg, Germany; 2 Department of Urology, Medical Center - University of Freiburg, Faculty of Medicine, University of Freiburg, Germany, Freiburg, Germany

**Keywords:** Outcomes research, Cancer

## Abstract

**Background:**

The Prostate Imaging Reporting and Data System, version 2.1 (PI-RADSv2.1) standardizes reporting of multiparametric MRI of the prostate. Assigned assessment categories are a risk stratification algorithm, higher categories indicate a higher probability of clinically significant cancer compared to lower categories. PI-RADSv2.1 does not define these probabilities numerically. We conduct a systematic review and meta-analysis to determine the cancer detection rates (CDR) of the PI-RADSv2.1 assessment categories on lesion level and patient level.

**Methods:**

Two independent reviewers screen a systematic PubMed and Cochrane CENTRAL search for relevant articles (primary outcome: clinically significant cancer, index test: prostate MRI reading according to PI-RADSv2.1, reference standard: histopathology). We perform meta-analyses of proportions with random-effects models for the CDR of the PI-RADSv2.1 assessment categories for clinically significant cancer. We perform subgroup analysis according to lesion localization to test for differences of CDR between peripheral zone lesions and transition zone lesions.

**Results:**

A total of 17 articles meet the inclusion criteria and data is independently extracted by two reviewers. Lesion level analysis includes 1946 lesions, patient level analysis includes 1268 patients. On lesion level analysis, CDR are 2% (95% confidence interval: 0–8%) for PI-RADS 1, 4% (1–9%) for PI-RADS 2, 20% (13–27%) for PI-RADS 3, 52% (43–61%) for PI-RADS 4, 89% (76–97%) for PI-RADS 5. On patient level analysis, CDR are 6% (0–20%) for PI-RADS 1, 9% (5–13%) for PI-RADS 2, 16% (7–27%) for PI-RADS 3, 59% (39–78%) for PI-RADS 4, 85% (73–94%) for PI-RADS 5. Higher categories are significantly associated with higher CDR (*P* < 0.001, univariate meta-regression), no systematic difference of CDR between peripheral zone lesions and transition zone lesions is identified in subgroup analysis.

**Conclusions:**

Our estimates of CDR demonstrate that PI-RADSv2.1 stratifies lesions and patients as intended. Our results might serve as an initial evidence base to discuss management strategies linked to assessment categories.

## Introduction

Multiparametric MRI of the prostate has emerged as the imaging modality of choice for the diagnosis of prostate cancer, being utilized in primary diagnosis [1], active surveillance [2], and relapse diagnosis [3]. In the setting of primary diagnosis, prostate MRI is interpreted according to the Prostate Imaging Reporting and Data System (PI-RADS), developed by the European Society of Urogenital Radiology (ESUR) and the American College of Radiology (ACR) [4, 5]. The PI-RADS lexicon is intended as a living document [6], meaning that the interpretation system is adapted as evidence about the diagnostic performance is generated. In 03/2019, the current version 2.1 replaced version 2.0, which had been established in 2015.

PI-RADS requires the interpreting radiologist to assign assessment categories to observed lesions. These categories range from one (clinically significant cancer is highly unlikely) over three (clinically significant cancer is equivocal) to five (clinically significant cancer is highly likely). The entire examination is assigned an overall assessment category, which equals the highest assigned lesion assessment category. A multitude of studies has validated this semantic risk stratification algorithm for version 2.0 – higher PI-RADSv2.0 categories are associated with higher rates of malignancy [7, 8].

In its current edition, PI-RADS does not provide numerical definitions of expected cancer detection rates of the assessment categories. Furthermore, no management recommendations are linked to the assessment categories. Both points have been realized in another reporting system developed by the American College of Radiology: the Breast Imaging Reporting and Data System (BI-RADS) [9]. BI-RADS has been established in 1997, and its development might serve as a model for the future of PI-RADS. It is stated in the current PI-RADS version that “specific recommendations and/or algorithms regarding biopsy and management will be included in future versions of PI-RADS” [5].

Precise estimates of expected cancer detection rates of the assessment categories are crucial to define adequate management recommendations. In addition, knowledge is required about the variability of cancer detection rates in the assessment categories to identify potentially problematic categories (with a high between-studies heterogeneity). The recognition of problematic assessment categories might lead to further refinement of the reporting lexicon.

Therefore, the aim of this systematic review and meta-analysis is to estimate the cancer detection rates of clinically significant prostate cancer of the PI-RADSv2.1 assessment categories. Following the PICOS criteria [10], we evaluate treatment naïve patients with suspicion for clinically significant prostate cancer (P), with prostate MRI reading according to PI-RADv2.1 as index test (I) and histopathological information as ground truth (C). Outcome (O) is defined as the cancer detection rate of the respective PI-RADSv2.1 assessment category. We consider full research articles reporting on retrospective or prospective cohorts (S) as eligible.

## Materials and methods

This systematic review reports items as recommended by the Preferred Reporting Items for Systematic reviews and Meta-Analyses (PRISMA) [10].

### Eligibility criteria, information sources, and search algorithm

Studies are considered eligible for this systematic review if they report on the diagnostic performance of the PI-RADSv2.1 assessment categories in treatment naïve patients. This restriction is applied because PI-RADS is explicitly intended to detect cancer in treatment naïve patients [5], i.e., patients that have not undergone surgery of the prostate, focal therapy, radiation therapy, or androgen-deprivation therapy. Reporting of a subset of categories is considered eligible, diagnostic performance is defined as both the reporting on distribution of PI-RADSv2.1 categories and histopathological information. The analysis needs to be on lesion level and/or patient level. Included studies perform the MRI reading blinded to the histopathological reference standard. We require the cohorts of included studies to be consecutive. We consider retrospective and prospective designs as eligible. Included studies have to be written in English.

**Fig. 1:**
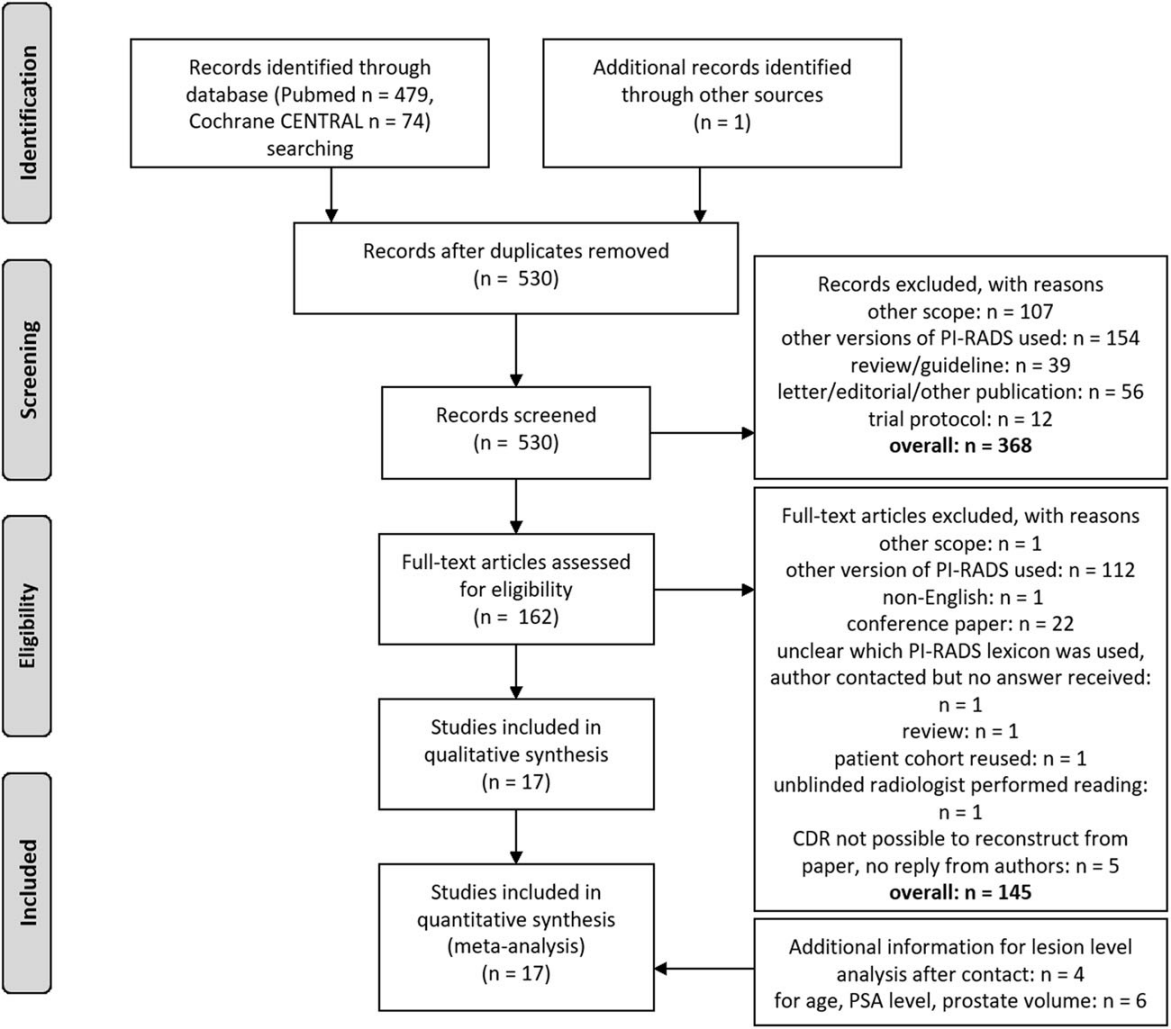
PRISMA flowchart of study selection for the systematic review and meta-analysis. Figure adapted from [11].

We perform a systematic PubMed search on 11/06/2020 [query: ((PI-RADS) OR (PIRADS) OR (PI RADS) OR (“prostate imaging reporting and data system”) OR (“prostate imaging: reporting and data system”)) AND (“2019/03/01” [Date - Publication]: “3000” [Date - Publication])]. Studies published prior to 04/2019 are not considered, since PI-RADSv2.0 has been the current interpretation system up to this point and PI-RADSv2.1 had not been published yet. Retrospective studies that include patients examined prior to 04/2019 are considered eligible when re-reading is performed according to PI-RADSv2.1. We additionally search the Cochrane Central Register of Controlled Trials (CENTRAL) [query: (PI-RADS):ti,ab,kw OR (PIRADS):ti,ab,kw OR (PI RADS):ti,ab,kw OR (prostate imaging reporting and data system):ti,ab,kw] with the same time restriction. Reference lists of included studies are screened for suitable publications not identified by the systematic search. Reasons for exclusion of studies are: (a) different scope, i.e., studies that do not address the diagnostic performance of PI-RADSv2.1 (b) other versions of PI-RADS (v1 or v2.0) have been employed for image interpretation, (c) no original research article (reviews, guidelines, letters, editorials, trial protocols, other), (d) unblinded reading of MRI, (e) not possible to reconstruct cancer detection rates from manuscript and authors do not reply despite being contacted twice. The PRISMA flowchart [11] for study selection is presented in Fig. 1.

### Data collection process and data items

Two independent observes (BO and MB) evaluate studies for eligibility, in case of discrepancy a consensus is reached by discussion. The same observers extract information from the selected studies with help of a predefined electronic datasheet. If a study reports on the performance of multiple readers, we extract information from the most experienced reader. After full information extraction, results are manually compared. Discrepancies are resolved by again accessing the original manuscripts and discussion.

If this essential information is not provided in the manuscript, but the manuscript reports on the diagnostic performance of PI-RADSv2.1 (for our definition, compare for section 2.1), corresponding authors are contacted twice and asked for the missing information. In addition, we ask for information of patient age, prostate-specific antigen level and prostate volume, if missing.

### Risk of bias assessment

Two independent observers (BO and MB) evaluate the risk of bias by employing the Quality Assessment of Diagnostic Accuracy Studies-2 (QUADAS-2) tool for the individual studies [12]. After full evaluation results are compared. Discrepancies are resolved by discussion.

### Definition of outcomes

The primary outcome of this systematic review is the detection rate of clinically significant prostate cancer (as defined in the single studies) in the PI-RADSv2.1 assessment categories on lesion level and patient level. The secondary outcome is the detection rate of any cancer (clinically significant and insignificant cancer combined) on lesion level and patient level. On the lesion level, the cancer detection rate is defined as the number of lesions with clinically significant cancer divided by the overall number of lesions in a certain PI-RADSv2.1 assessment category. On the patient level, the cancer detection rate is defined as the number of patients with clinically significant cancer divided by the overall number of patients in a certain PI-RADSv2.1 assessment category. In diagnostic accuracy studies, this statistic is generally referred to as positive predictive value.

### Data synthesis and statistical analysis

We derive pooled estimates and 95% confidence intervals of the cancer detection rates of the PI-RADSv2.1 assessment categories with random-effects meta-analyses of proportions [13]. Since the inverse variance method for estimation of confidence intervals is problematic for values close to 0 or 1 (and such values can be expected for PI-RADS assessment categories 1, 2, and 5), we use the double arcsine transformation of proportions [13]. Heterogeneity of cancer detection rates between studies is investigated with the I^2^ statistic [14]. I^2^ measures the relative amount of variation between studies beyond what can be expected due to chance alone [14], values range between 0% (no heterogeneity) and 100% (maximum heterogeneity). We consider an I^2^ > 50% to denote considerable heterogeneity. We also report the between-study variance (τ^2^, DerSimonian-Laird estimator) of the random-effects models as a quantitative absolute estimator of the extent of heterogeneity.

The positive predictive value of a diagnostic test is, unlike sensitivity and specificity, directly dependent upon disease prevalence [15]. Given a fixed sensitivity and specificity, the resulting relationship is non-linear [15]. We, therefore, correlate (Spearman correlation) disease prevalence with the cancer detection rates of the single PI-RADSv2.1 assessment categories as reported in the individual studies. We define pretest probability in the individual study as the ratio of patients/lesions with clinically significant cancer divided by the number of all included patients/lesions, respectively. A predefined subgroup analysis (stratified by localization of the lesion, peripheral zone versus transition zone) is planned for the lesion level analysis, summary measures are compared with the chi-squared statistic. We test for a significant dependence of cancer detection rate from the assessment category with univariate meta-regression (mixed-effects models). A *P* < 0.05 is considered to denote a statistically significant difference/dependence.

Possible publication bias is graphically examined by inspection of funnel plots [16]. Following the recommendation of Hunter et al., we plot study size on the *y* axis instead of standard error [17]. Egger’s test is employed for analyses with ≥ 10 studies to test for asymmetry [18].

## Results

**Fig. 2:**
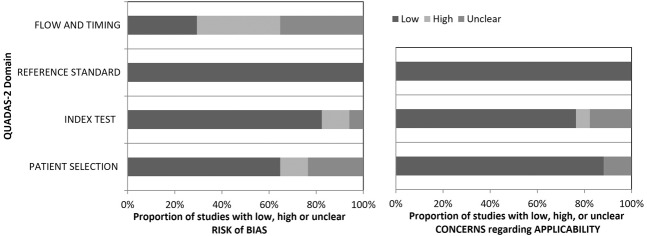
Summary of QUADAS-2 evaluation of included studies. Figure adapted from [40].

The characteristics of the finally included 17 studies [19–35] are given in Table 1, technical MRI specifications employed in the individual studies are given in Supplementary Table 1, the summary QUADAS-2 evaluation of included studies is presented in Fig. 2. Mean PSA level ranges between 7.2 ng/ml and 21.72 ng/ml, median PSA level ranges between 5.79 ng/ml and 11.7 ng/ml. Mean age ranges between 63.1 years and 69.8 years, the median age ranges between 66 years and 69 years. Overall, we include information from 1946 histopathologically verified lesions in the lesion level analysis and information from 1268 patients in the patient level analysis.

**Table 1:** General information of analyzed studies.

Author (ref. No)	Year	Patient recruitment	Study type	Patient/lesion level analysis	Number of patients	Number of patients with insign. CA	Number of patients with sign. CA	Number of lesions	Number of lesion with insign. CA	Number of lesions with sign. CA	Definition sign. CA	Includes patient under active surveillance	PSA mean/median	Age mean/median	Prostate volume mean/median	Previous biopsy status (biopsy-naive, prior-negative biopsy, mixed, not defined)	Verification	Biopsy technique
Bao [19]	2020	01/2018–12/2019	retrospective	patient level analysis	638	32	287	NR	NR	NR	Gleason Score ≥ 7a	NR	21.72/11.7	NR/69	NR/NR	not defined	lesion + systematic biopsy	mixed
Brancato [20]	2020	04/2013–09/2018	retrospective	lesion level analysis	111	34	38	117	37	41	Gleason Score ≥ 7a	NR	11.29/8	NR/69	57.5/NR	not defined	lesion + systematic biopsy	mixed
Byun [21]	2020	01/2018–06/2018	retrospective	lesion level analysis	142	21	121	201	13	70	Gleason score ≥ 7a and/or a volume ≥ 0.5 cm^3^, and/or extracapsular extension according to PI-RADS v2	no	8.33/NR	67/NR	32.83/NR	not defined	prostatectomy	RP
Costa [22]	2020	04/2019–04/2020	retrospective	lesion level analysis	103	14	10	110	14	10	Gleason Score ≥ 7a	yes	NR/NR	NR/NR	NR/NR	not defined	mixed	mixed
Falagario [23]	2020	03/2013–05/2017	retrospective	patient level analysis	451	76	211	NR	NR	NR	Gleason Score ≥ 7a	no	NR/NR	NR/NR	NR/NR	no biopsy 6 months prior to MRI	lesion + systematic biopsy	mixed
Gorin [24]	2019	02/2018–06/2019	mixed^a^	lesion level analysis	95	27	NR	124	27	52	Gleason Score ≥ 7a	yes	NR/6.9	NR/68.8	36/NR	not defined	lesion + systematic biopsy	US cognitive
Han [25]	2020	06/2010–05/2017	retrospective	patient level analysis	123	13	37	NR	NR	NR	Gleason Score ≥ 7a	NR	7.2/NR	66.3/NR	NR/NR	not defined	lesion + systematic biopsy	US cognitive
Hosseiny [26]	2020	02/2014–07/2018	retrospective	patient level analysis	79	6	30	99	6	33	Gleason Score ≥ 7a	no	NR/8.6	64.1/NR	NR/54	prior-negative biopsy	lesion biopsy	in-bore
HosseinyB [27]	2020	02/2012–03/2019	retrospective	lesion level analysis	379	69	193	475	97	208	Gleason score ≥ 7a	yes (separate analysis)	NR/NR	NR/NR	NR/NR	mixed	lesion biopsy	in-bore
Hötker [28]	2020	01/2015–12/2017	retrospective	patient level analysis	229	26	122	NR	NR	NR	Gleason score ≥ 7a	NR	8.2/NR	63.1/NR	50.73/46.44	not defined	lesion + systematic biopsy	MRI-US fusion transperineal
Lim [29]	2020	01/2015–07/2018	retrospective	lesion level analysis	104	NR	NR	109	21	14	Gleason Score ≥ 7a	NR	10.6/NR	64.8/NR	71/NR	not defined	lesion biopsy	US cognitive
Rudolph [30]	2020	01/2012–07/2015	retrospective	lesion level analysis	333	64	152	359	58	135	Gleason score ≥ 7a	NR	12.8/NR	66.8/NR	62.8/NR	not defined	lesion + systematic biopsy	MRI-US fusion transrectal
Tamada [31]	2021	03/2019–01/2020	retrospective	lesion level analysis	103	NR	NR	165	NR	81	Gleason Score ≥ 7a and tumor diameter ≥ 5 mm, or Gleason Score = 3 + 3 and tumor size ≥ 0.5 mL (tumor diameter ≥ 8 mm)	NR	NR/6.92	69.8/NR	NR/NR	not defined	mixed	mixed
Vilanova [32]	2020	07/2019–03/2020	prospective	lesion level analysis	30	3	19	30	3	19	Gleason Score ≥ 7a or maximum cancer core length > 3 mm for Gleason 6	NR	13.1/NR	66/NR	47.2/NR	mixed	lesion biopsy	in-bore
Walker [33]	2020	04/2019–09/2019	prospective	both	110	16	43	171	17	57	Gleason score ≥ 7a	NR	NR/5.79	NR/66	NR/55.5	not defined	lesion + systematic biopsy	MRI-US fusion transrectal
Wang [34]	2020	03/2016–10/2018	retrospective	patient level analysis	584	NR	111	NR	NR	NR	Gleason Grade Group ≥ 1 However, distribution of Gleason grade groups is provided in supplement, we use ≥7a	NR	NR/NR	NR/NR	NR/NR	not defined	lesion biopsy	MRI-US fusion transrectal
Xu [35]	2020	01/2017–12/2017	retrospective	lesion level analysis	85	2	25	85	2	25	Gleason Score ≥ 7a and/or volume > 0.5 mL, and/or extraprostatic extension	NR	NR/9.08	67.85/NR	56.85/NR	not defined	systematic only, saturation biopsy	transperineal saturation

*NR* not reported

^a^Not reported whether blinded re-reading was performed, it is explicitly stated that MRI studies were graded using PI-RADSv2.1.

### Detection of clinically significant cancer

**Fig. 3:**
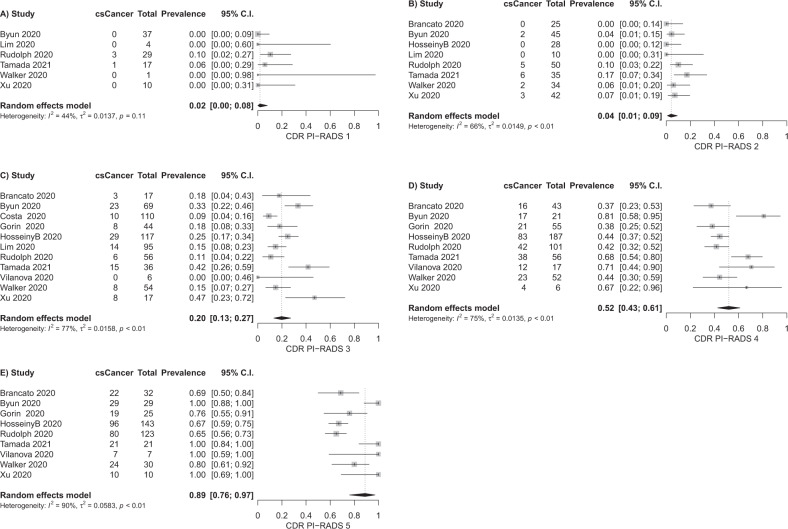
Forest plots of lesion level analysis, cancer detection rates of PI-RADSv2.1 assessment categories for clinically significant cancer as outcome variable. **A** PI-RADS 1, (**B**) PI-RADS 2, (**C**) PI-RADS 3, (**D**) PI-RADS 4, (**E**) PI-RADS 5.

For the detection of clinically significant cancer, information from 11 studies can be used for the lesion level analysis and information from seven studies can be used for the patient level analysis. The forest plots for the single PI-RADSv2.1 assessment categories and derived pooled estimates are given in Fig. 3 (lesion level) and Supplementary Fig. 1 (patient level). Lesion level analysis results in pooled cancer detection rates of 2% for PI-RADS 1, 4% for PI-RADS 2, 20% for PI-RADS 3, 52% for PI-RADS 4, 89% for PI-RADS 5. Patient level analysis results in pooled cancer detection rates of 6% for PI-RADS 1, 9% for PI-RADS 2, 16% for PI-RADS 3, 59% for PI-RADS 4, 85% for PI-RADS 5. For the 95% confidence intervals, refer to Fig. 3 and Supplementary Fig. 1. The association of higher PI-RADSv2.1 assessment categories with higher cancer detection rates is significant (*P* < 0.001 for both analyses). We observe considerable heterogeneity of results, with I^2^ values > 50% for PI-RADS 2–5 in the lesion level analysis, and I^2^ values > 50% for PI-RADS 1 and 3–5 in the patient level analysis.

### Detection of any cancer

For the detection of any cancer (combination of clinically insignificant and significant cancer), information from nine studies can be used for the lesion level analysis and information from six studies can be used for the patient level analysis. The forest plots for the single PI-RADSv2.1 assessment categories and derived pooled estimates are given in Supplementary Fig. 2 (lesion level) and Supplementary Fig. 3 (patient level). Lesion level analysis results in pooled cancer detection rates of 3% for PI-RADS 1, 9% for PI-RADS 2, 34% for PI-RADS 3, 70% for PI-RADS 4, 97% for PI-RADS 5. Patient level analysis results in pooled cancer detection rates of 32% for PI-RADS 1, 17% for PI-RADS 2, 27% for PI-RADS 3, 77% for PI-RADS 4, 97% for PI-RADS 5. For the 95% confidence intervals, refer to Supplementary Figs. 2 and 3. The association of higher PI-RADSv2.1 assessment categories with higher cancer detection rates is significant (*P* < 0.001 for both analyses). Notably, the patient level analysis for any cancer includes data from only six studies, the estimate for PI-RADS 1 is derived from data of two relatively small samples (Supplementary Fig. 3). We observe considerable heterogeneity of results, with I^2^ values > 50% for PI-RADS 1–4 in the lesion level analysis, and I^2^ values > 50% for PI-RADS 1–5 in the patient level analysis.

### Cancer detection rate stratified by lesion localization

Our subgroup analysis according to lesion localization (Supplementary Fig. 4) shows that estimates of cancer detection rates do not differ for PI-RADS 2–5 between peripheral zone and transition zone (*P* > 0.05 each). For PI-RADS 1 (*P* = 0.04), only 1 study reports lesions in the peripheral zone for this assessment category – the obtained difference in cancer detection rate in PI-RADS category 1 should therefore be considered with care.

### Dependence of cancer detection rate from pretest probability

The dependence of cancer detection rate (clinically significant cancer) from pretest probability is presented in Supplementary Fig. 5. Size of the data points is set proportional to sample size. Spearman correlation of pretest probability with cancer detection rate is 0.78/0.5 for PI-RADS 1, 0.32/0.8 for PI-RADS 2, 0.07/0.49 for PI-RADS 3, 0.17/0.4 for PI-RADS 4 and −0.04/0.8 for PI-RADS 5 (lesion level/patient level). Fewer data points are used for estimation of correlation in the patient level analysis, and the resulting strong correlation coefficients have to be interpreted with care. Pretest probability ranges between 0.3 and 0.5 in the majority of studies that include PI-RADS 4 and 5 lesions, and estimates of cancer detection rate scatter around the pooled estimates in this range. Three studies can be regarded as outliers regarding pretest probability. Vilanova et al. (30 lesions with 19 clinically significant cancers, only PI-RADS 3, 4, and 5 lesions considered) report rather high cancer detection rates for PI-RADS 4 and 5 [32]. Lim et al. (109 lesions, 14 clinically significant cancers, only PI-RADS 1, 2, and 3 lesions considered) report a low cancer detection rate of PI-RADS 2 (0%, 10 lesions) [29]. Costa et al. (110 lesions with 10 clinically significant cancers, only PI-RADS 3 lesions considered) report a relatively low cancer detection rate of PI-RADS 3 [22]. This might partly be explained with the high/low pretest probability in the respective studies.

### Risk of bias evaluation

From Supplementary Figs. 6 and 7 (funnel plots for lesion level and patient level analysis) we do not infer a systematic publication bias for cancer detection rate estimates—study estimates scatter symmetrically around the summary measure in the majority of cases. Our QUADAS-2 analysis (Fig. 2) demonstrates a considerable proportion of studies with unclear and high risk in the domains flow and timing and patient selection. Eight studies did not report on the time between MRI and performed biopsy. Six studies did not report inclusion and exclusion criteria or had exclusion criteria that possibly led to bias. In addition, some studies employed different biopsy techniques for lesion verification (compare for Table 1).

## Discussion

In our systematic review and meta-analysis, we present initial estimates of the cancer detection rates of the PI-RADSv2.1 assessment categories on lesion level and patient level. Overall, the assessment categories perform as intended, with higher categories having higher cancer detection rates. At the moment, PI-RADS assessment categories are defined semantically to have very low (PI-RADS 1) to very high (PI-RADS 5) probability for the presence of clinically significant cancer [5]. PI-RADSv2.1 does neither provide exact numerical values of the cancer detection rates of the single categories nor probability ranges. Since it is explicitly stated that “specific recommendations and/or algorithms regarding biopsy and management will be included in future versions of PI-RADS” [5], robust estimates of the cancer detection rates of the assessment categories are required.

A systematic review and meta-analysis of the cancer detection rates of the PI-RADSv2.0 lexicon has recently been published [8]. In their work, Mazzone et al. report detection rates of 8% for PI-RADS 2, 13% for PI-RADS 3, 40% for PI-RADS 4 and 69% for PI-RADS 5 for clinically significant cancer on an index-lesion level [8], with an overall lower cancer detection rate on lesion level (31% versus 40%) [8]. These results are close to our reported cancer detection rates for PI-RADSv2.1 on lesion level with 2% for PI-RADS 1, 4% for PI-RADS 2, 20% for PI-RADS 3 and 52% for PI-RADS 4. For PI-RADS 5, we report a higher pooled summary measure with 89%.

The changes from PI-RADSv2.0 to PI-RADSv2.1 predominantly affect transition zone categories 1–3 [5]. Typical, completely encapsulated BPH nodules, now scored as PI-RADS category 1, are distinguished from atypical nodules. These may be upgraded from PI-RADS category 2 to PI-RADS category 3 based on their signal intensity on highly diffusion weighted images and corresponding ADC maps. Furthermore, the wording for interpretation of diffusion weighted images for categories 2 and 3 has been sharpened [5]. It is possible that differences between the estimates of Mazzone et al. and the results presented in our work reflect these changes for assessment categories 2 and 3. The point that Mazzone et al. report cancer detection rates on index-lesion level and our lesion level analysis includes all lesions reported in the single studies might also contribute to differences. Furthermore, 95% confidence intervals for our estimates for assessment categories 2 and 3 (1–9% for category 2, 13–27% for category 3, Fig. 3) overlap with the corresponding confidence intervals reported in the work by Mazzone et al. (4–14% for category 2, 10–17% for category 3) [8]. The same holds true for comparison with our reported cancer detection rates on patient level (Supplementary Fig. 1). Given this evidence, we cannot infer that a significant change in cancer detection rates of these assessment categories has been introduced with PI-RADSv2.1. Differences in the summary measures of cancer detection rate can either be due to chance, due to a difference in the interpretation lexicon, or due to differences between the study populations examined.

PI-RADS assessment categories 4 and 5 have been shown to have a high probability of clinically significant cancer in version 2.0 [7, 8], our results corroborate this for version 2.1. The standard management recommendation is to refer patients rated PI-RADS 4 or 5 to biopsy [1], with biopsy strategy depending on the clinical context [1]. For PI-RADS assessment category 3, this management is even more flexible. Biopsy and follow-up imaging can be advocated in these cases, depending on the clinical context [1, 36]. Lesions in assessment category 3 comprise different entities in the transition zone, i.e., upgraded atypical nodules and lesions with obscured margins in T2w [5]. Costa et al. perform a direct comparison of these two entities, with a cancer detection rate of 6% for upgraded atypical nodules and 11% for conventional PI-RADS 3 lesions in the transition zone [22], the difference being not statistically significant. Likewise, Lim et al. report a lower cancer detection rate for upgraded atypical nodules (8%) compared to conventional PI-RADS 3 lesions (20%) [29]. Conversely, Byun et al. report a higher cancer rate in upgrade nodules compared to conventional PI-RADS 3 lesions [21]. Overall, we do not observe a difference of cancer detection rate between PI-RADS 3 lesions in the peripheral zone and transition zone (Supplementary Fig. 4, *P* = 0.52). Our analysis pools upgraded nodules and conventional PI-RADS 3 lesions in the transition zone into one category.

The width of the reported confidence intervals for cancer detection rates highlights the heterogeneity of included studies. Several limitations impair the generalizability of our results: heterogeneity of study populations, only a few studies available for patient level analysis, different definitions of histopathological reference especially on patient level and a possible verification bias for low assessment categories. We will discuss these limitations in the following paragraphs.

First, patient cohorts in our study are not homogeneous. Following Table 1, there is heterogeneity and uncertainty according to inclusion of patients under active surveillance (11 not reported, 3 yes, 3 no) and prior biopsy status (13 not reported, 1 prior negative biopsy, 2 mixed, 1 without biopsy 6 months prior to MRI). The composition of the study population impacts cancer detection rates. In the study by Hosseiny et al., detection rates of categories 3–5 are lower in patients with prior negative biopsy compared to patients under active surveillance and biopsy naïve patients [27]. This dependence is also reported in the meta-analysis by Mazzone et al. for PI-RADSv2.0: in patients with prior negative biopsy the overall positive predictive value is 32%, compared to 42% in biopsy naïve patients [8]. Second, the standard of reference is heterogeneously defined in the included studies. From Table 1 it follows that the majority of studies treats any occurrence of Gleason score ≥ 7a as clinically significant cancer, whereas the minority adheres to the more detailed and more difficult to establish PI-RADS definition (“pathology/histology as Gleason score ≥ 7, including 3 + 4 with prominent but not predominant Gleason 4 component, and/or volume ≥ 0.5cc, and/or extraprostatic extension”) [5]. We follow Mazzone et al. in pooling data despite this issue [8]. Third, pretest probability varies across studies (for the majority of studies it ranges between 0.3 and 0.5, compare for Supplementary Fig. 5). If we consider pretest probability as a surrogate parameter for patient spectrum, this also hints at the application of prostate MRI in different clinical settings across the included studies. Our meta-analysis of cancer detection rates is based on fewer studies compared to the work of Mazzone et al. for PI-RADSv2.0—which precludes robust subgroup analyses according to the aforementioned variables. We thus consider our estimates to reflect the heterogeneity of current clinical practice. We highlight that the estimation of pretest probability as defined in section 2.5. is necessarily low for studies that only include lesions/patients with PI-RADS assessment category ≤3 [22, 29] – since most malignant cases of the patient cohort are to be expected in categories 4 and 5, and these are not reported in the respective studies.

Our patient level analysis includes fewer studies compared to lesion level analysis (Fig. 3 and Supplementary Fig. 1). Moreover, patient level analysis is aggravated due to the different histopathological reference standards of the included studies. For example, Hötker et al. combine a transperineal template saturation biopsy with additional targeted biopsies [28]. Bao et al. use the information from systematic biopsy for part of the cohort and information from radical prostatectomy for the other part [19]. Hosseiny et al. report on patient level and employ the information of in-bore biopsy only as a reference standard, with the additional inclusion criterion of a negative 12x systematic biopsy up to one year prior to MRI [26]. Falagario et al. use the information of 12x systematic biopsy and additional targeted biopsy as a reference standard [23]. Larger, more homogenous studies regarding outcome definition are warranted to derive more robust estimates for cancer detection rates of the PI-RADSv2.1 assessment categories on patient level.

We define any kind of histopathology as reference standard in our study in accordance with previous systematic reviews and meta-analyses of PI-RADS [8, 37, 38]. This facilitates the comparison of our results to estimates of cancer detection rates of PI-RADSv2.0. The cancer detection rates of categories 4 and 5 are most likely not biased because of this, since biopsy is the generally established management recommendation [1]. Especially for categories 1 and 2, this is not the case. Inclusion of only histopathologically verified PI-RADS 1 and 2 lesions/patients might have introduced verification bias [39] – the majority of PI-RADS 1 and 2 cases will not undergo biopsy. The histopathologically verified cases cannot be considered a random sample from all PI-RADS 1 and 2 cases, the clinical context indicated biopsy despite an assigned low probability for clinically significant cancer. We therefore can expect the true cancer detection rates of PI-RADS 1 and 2 (and to some degree PI-RADS 3) to be lower than the reported estimates.

As expected, we report higher detection rates for any cancer compared to clinically significant cancer on both, lesion level and patient level (Supplementary Figs. 2, 3). Although PI-RADS does not intend to detect clinically insignificant cancer, knowledge of expected detection rates seems worthwhile in respect to patient communication and management considerations (expected rate of overdiagnosis).

To conclude, in this systematic review and meta-analysis we provide estimates of the cancer detection rates of the PI-RADSv2.1 assessment categories on lesion level and patient level. As intended, higher categories are associated with a higher probability for clinically significant cancer on both, lesion level and patient level. Our estimates might serve as an initial evidence base for discussion of management strategies linked to assessment categories—which is planned in future version of PI-RADS. Given our results, we believe that biopsy will remain the standard management for PI-RADS 4 and 5 cases. In case of PI-RADS 3, cost-benefit analyses seem appropriate to define management strategies in different clinical scenarios, taking into account prior biopsy status, patient age, comorbidities and potentially further diagnostic variables like PSA density. Further studies addressing the diagnostic accuracy and cancer detection rates of the assessment categories, especially of category 3, are required to obtain robust estimates for different clinical scenarios.

## Supplementary information

Supplemental material
