# Supplementary material for: Cancer detection rates of the PI-RADSv2.1 assessment categories: systematic review and meta-analysis on lesion level and patient level
Source: Prostate Cancer Prostatic Dis. 2021 Jul 6;25(2):256–63. doi: 10.1038/s41391-021-00417-1 (PMC9184264; doi:10.1038/s41391-021-00417-1)
Supplement: Supplementary file 1 — Supplemental material [file 41391_2021_417_MOESM1_ESM.pdf]

Supplementary figure 1 (patient level, outcome: cs cancer)

A) Study	csCancer	Total	Prevalence	95% C.I.
Han 2020	0	8	0.00	[0.00; 0.37]
Hötker 2020	2	34	0.06	[0.01; 0.20]
Walker 2020	3	16	0.19	[0.04; 0.46]
**Random effects model**			**0.06**	**[0.00; 0.20]**

Heterogeneity: $I^2 = 55\%$, $\tau^2 = 0.0187$, $p = 0.11$

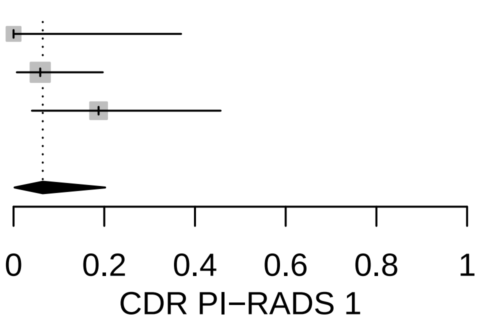

B) Study	csCancer	Total	Prevalence	95% C.I.
Bao 2020	22	260	0.08	[0.05; 0.13]
Han 2020	3	57	0.05	[0.01; 0.15]
Hosseiny 2020	0	2	0.00	[0.00; 0.84]
Hötker 2020	7	45	0.16	[0.06; 0.29]
**Random effects model**			**0.09**	**[0.05; 0.13]**

Heterogeneity: $I^2 = 21\%$, $\tau^2 = 0.0012$, $p = 0.28$

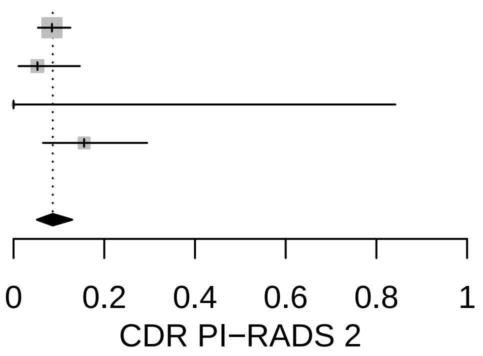

C) Study	csCancer	Total	Prevalence	95% C.I.
Bao 2020	16	72	0.22	[0.13; 0.34]
Falagario 2020	8	81	0.10	[0.04; 0.19]
Han 2020	3	10	0.30	[0.07; 0.65]
Hosseiny 2020	6	38	0.16	[0.06; 0.31]
Hötker 2020	8	25	0.32	[0.15; 0.54]
Wang 2020	4	136	0.03	[0.01; 0.07]
**Random effects model**			**0.16**	**[0.07; 0.27]**

Heterogeneity: $I^2 = 84\%$, $\tau^2 = 0.0246$, $p < 0.01$

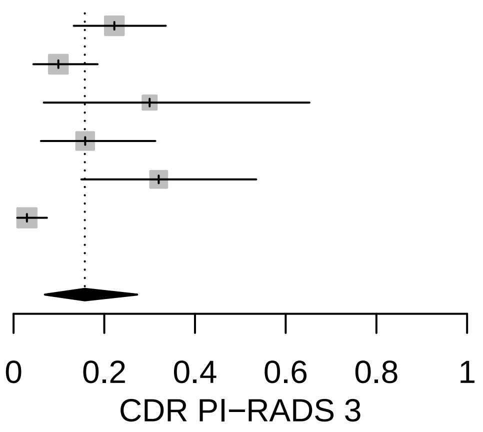

D) Study	csCancer	Total	Prevalence	95% C.I.
Falagario 2020	32	75	0.43	[0.31; 0.55]
Han 2020	16	29	0.55	[0.36; 0.74]
Hosseiny 2020	13	23	0.57	[0.34; 0.77]
Hötker 2020	72	91	0.79	[0.69; 0.87]
**Random effects model**			**0.59**	**[0.39; 0.78]**

Heterogeneity: $I^2 = 88\%$, $\tau^2 = 0.0377$, $p < 0.01$

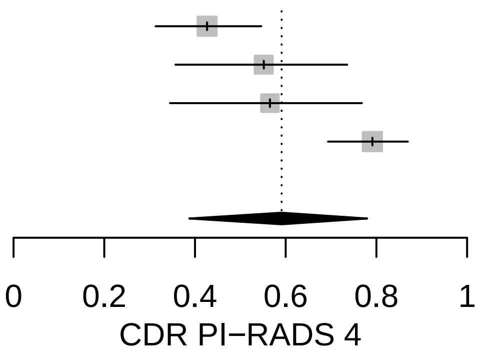

E) Study	csCancer	Total	Prevalence	95% C.I.
Falagario 2020	165	197	0.84	[0.78; 0.89]
Han 2020	15	19	0.79	[0.54; 0.94]
Hosseiny 2020	11	16	0.69	[0.41; 0.89]
Hötker 2020	33	34	0.97	[0.85; 1.00]
**Random effects model**			**0.85**	**[0.73; 0.94]**

Heterogeneity: $I^2 = 70\%$, $\tau^2 = 0.0156$, $p = 0.02$

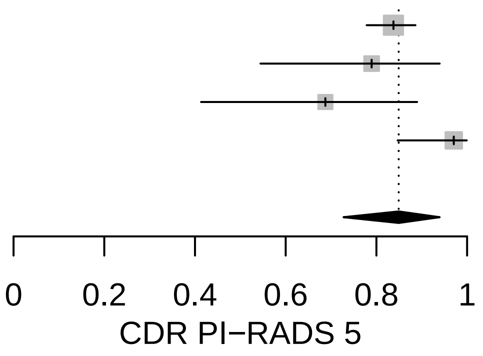

Supplementary figure 2 (lesion level, outcome: any cancer)

A)

Study	any Cancer	Total	Prevalence	95% C.I.
Byun 2020	6	37	0.16	[0.06; 0.32]
Lim 2020	0	4	0.00	[0.00; 0.60]
Walker 2020	0	1	0.00	[0.00; 0.98]
Xu 2020	0	10	0.00	[0.00; 0.31]
**Random effects model**			**0.03**	**[0.00; 0.20]**

Heterogeneity: $I^2 = 59\%$, $\tau^2 = 0.0463$, $p = 0.06$

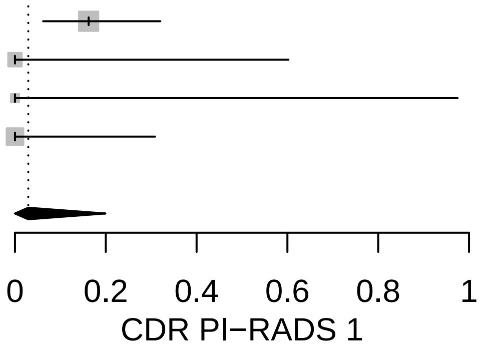

B)

Study	any Cancer	Total	Prevalence	95% C.I.
Brancato 2020	5	25	0.20	[0.07; 0.41]
Byun 2020	3	45	0.07	[0.01; 0.18]
HosseinyB 2020	2	28	0.07	[0.01; 0.24]
Lim 2020	0	10	0.00	[0.00; 0.31]
Walker 2020	7	34	0.21	[0.09; 0.38]
Xu 2020	3	42	0.07	[0.01; 0.19]
**Random effects model**			**0.09**	**[0.04; 0.17]**

Heterogeneity: $I^2 = 53\%$, $\tau^2 = 0.0093$, $p = 0.06$

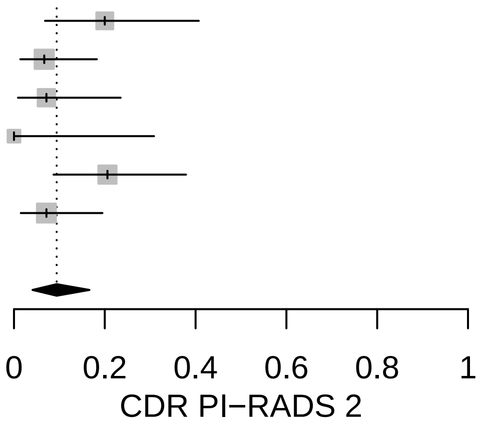

C)

Study	any Cancer	Total	Prevalence	95% C.I.
Brancato 2020	9	17	0.53	[0.28; 0.77]
Byun 2020	27	69	0.39	[0.28; 0.52]
Costa 2020	24	110	0.22	[0.15; 0.31]
Gorin 2020	16	44	0.36	[0.22; 0.52]
HosseinyB 2020	51	117	0.44	[0.34; 0.53]
Lim 2020	35	95	0.37	[0.27; 0.47]
Vilanova 2020	0	6	0.00	[0.00; 0.46]
Walker 2020	13	54	0.24	[0.13; 0.38]
Xu 2020	9	17	0.53	[0.28; 0.77]
**Random effects model**			**0.34**	**[0.26; 0.43]**

Heterogeneity: $I^2 = 74\%$, $\tau^2 = 0.0129$, $p < 0.01$

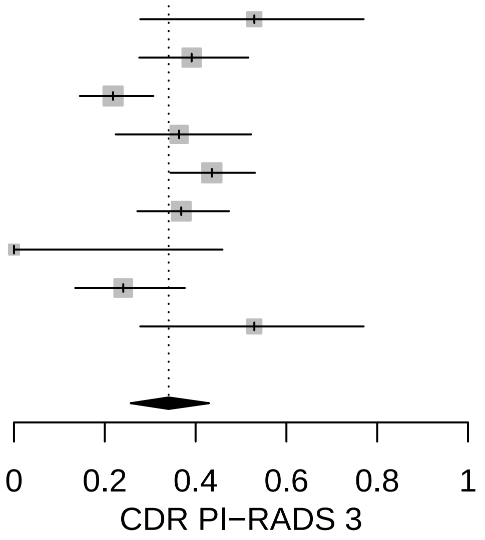

D)

Study	any Cancer	Total	Prevalence	95% C.I.
Brancato 2020	33	43	0.77	[0.61; 0.88]
Byun 2020	18	21	0.86	[0.64; 0.97]
Gorin 2020	34	55	0.62	[0.48; 0.75]
HosseinyB 2020	118	187	0.63	[0.56; 0.70]
Vilanova 2020	15	17	0.88	[0.64; 0.99]
Walker 2020	27	52	0.52	[0.38; 0.66]
Xu 2020	5	6	0.83	[0.36; 1.00]
**Random effects model**			**0.70**	**[0.61; 0.79]**

Heterogeneity: $I^2 = 68\%$, $\tau^2 = 0.0117$, $p < 0.01$

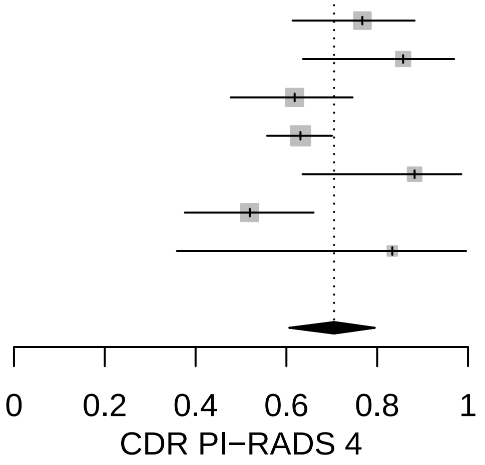

E)

Study	any Cancer	Total	Prevalence	95% C.I.
Brancato 2020	31	32	0.97	[0.84; 1.00]
Byun 2020	29	29	1.00	[0.88; 1.00]
Gorin 2020	23	25	0.92	[0.74; 0.99]
HosseinyB 2020	134	143	0.94	[0.88; 0.97]
Vilanova 2020	7	7	1.00	[0.59; 1.00]
Walker 2020	27	30	0.90	[0.73; 0.98]
Xu 2020	10	10	1.00	[0.69; 1.00]
**Random effects model**			**0.97**	**[0.92; 0.99]**

Heterogeneity: $I^2 = 47\%$, $\tau^2 = 0.0072$, $p = 0.08$

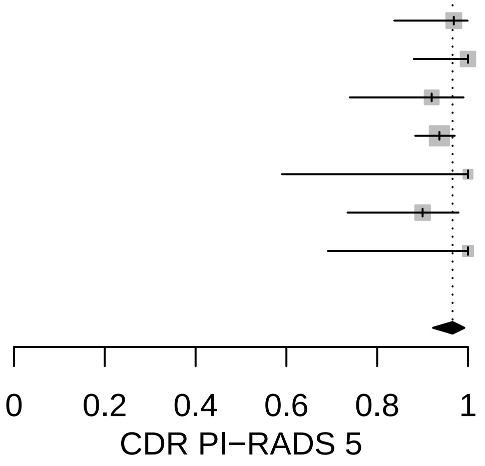

Supplementary figure 3 (patient level, outcome: any cancer)

A) Study	any Cancer	Total	Prevalence	95% C.I.
Hötker 2020	6	34	0.18	[0.07; 0.35]
Walker 2020	8	16	0.50	[0.25; 0.75]
**Random effects model**			**0.32**	**[0.06; 0.65]**

Heterogeneity: $I^2 = 81\%$, $\tau^2 = 0.0504$, $p = 0.02$

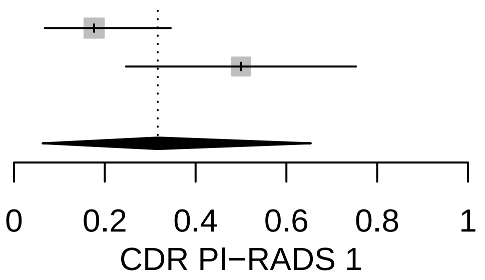

B) Study	any Cancer	Total	Prevalence	95% C.I.
Bao 2020	37	260	0.14	[0.10; 0.19]
Hosseiny 2020	0	2	0.00	[0.00; 0.84]
Hötker 2020	12	45	0.27	[0.15; 0.42]
**Random effects model**			**0.17**	**[0.08; 0.30]**

Heterogeneity: $I^2 = 60\%$, $\tau^2 = 0.0095$, $p = 0.08$

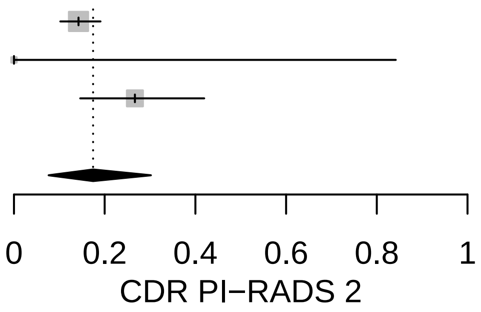

C) Study	any Cancer	Total	Prevalence	95% C.I.
Bao 2020	20	72	0.28	[0.18; 0.40]
Falagario 2020	28	81	0.35	[0.24; 0.46]
Hosseiny 2020	7	38	0.18	[0.08; 0.34]
Hötker 2020	13	25	0.52	[0.31; 0.72]
Wang 2020	18	136	0.13	[0.08; 0.20]
**Random effects model**			**0.27**	**[0.16; 0.40]**

Heterogeneity: $I^2 = 84\%$, $\tau^2 = 0.0200$, $p < 0.01$

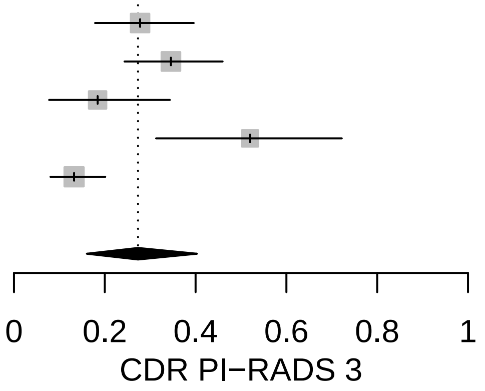

D) Study	any Cancer	Total	Prevalence	95% C.I.
Falagario 2020	52	75	0.69	[0.58; 0.79]
Hosseiny 2020	15	23	0.65	[0.43; 0.84]
Hötker 2020	83	91	0.91	[0.83; 0.96]
**Random effects model**			**0.77**	**[0.57; 0.93]**

Heterogeneity: $I^2 = 88\%$, $\tau^2 = 0.0326$, $p < 0.01$

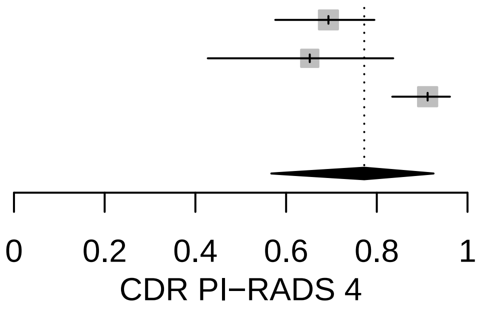

E) Study	any Cancer	Total	Prevalence	95% C.I.
Falagario 2020	190	197	0.96	[0.93; 0.99]
Hosseiny 2020	14	16	0.88	[0.62; 0.98]
Hötker 2020	34	34	1.00	[0.90; 1.00]
**Random effects model**			**0.97**	**[0.90; 1.00]**

Heterogeneity: $I^2 = 69\%$, $\tau^2 = 0.0135$, $p = 0.04$

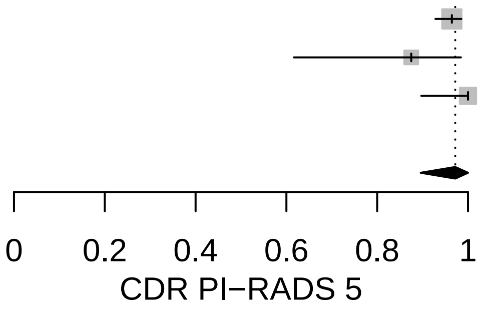

Supplementary figure 4 (lesion level, outcome: cs cancer)

A)

Study	csCancer	Total	Prevalence	95% C.I.
**localization = PZ**				
Rudolph 2020	2	13	0.15	[0.02; 0.45]
**Random effects model**			**0.15**	**[0.02; 0.39]**
Heterogeneity: not applicable				
**localization = TZ**				
Byun 2020	0	37	0.00	[0.00; 0.09]
Rudolph 2020	1	16	0.06	[0.00; 0.30]
Xu 2020	0	10	0.00	[0.00; 0.31]
**Random effects model**			**0.01**	**[0.00; 0.06]**
Heterogeneity: I^2 = 34%, τ^2 = 0.0074, p = NA				

Heterogeneity: I^2 = 62%, τ^2 = 0.0245, p = 0.05
Test for subgroup differences: χ^2_1 = 4.10, df = 1 (p = 0.04)

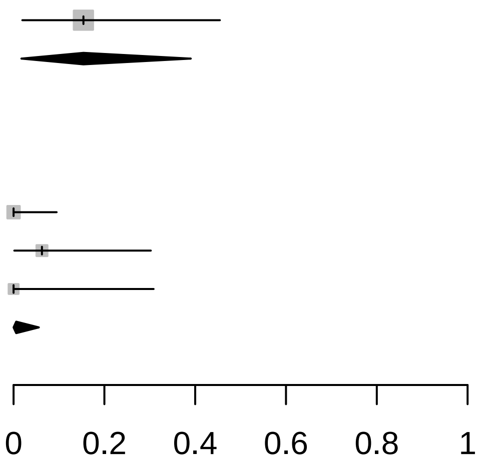

B)

Study	csCancer	Total	Prevalence	95% C.I.
**localization = PZ**				
Brancato 2020	0	24	0.00	[0.00; 0.14]
Rudolph 2020	3	23	0.13	[0.03; 0.34]
**Random effects model**			**0.03**	**[0.00; 0.27]**
Heterogeneity: I^2 = 84%, τ^2 = 0.0576, p = 0.89				
**localization = TZ**				
Brancato 2020	0	1	0.00	[0.00; 0.98]
Byun 2020	2	45	0.04	[0.01; 0.15]
Rudolph 2020	2	27	0.07	[0.01; 0.24]
Xu 2020	3	42	0.07	[0.01; 0.19]
**Random effects model**			**0.06**	**[0.02; 0.11]**
Heterogeneity: I^2 = 0%, τ^2 = 0, p = 0.01				

Heterogeneity: I^2 = 34%, τ^2 = 0.0052, p = 0.18
Test for subgroup differences: χ^2_1 = 0.11, df = 1 (p = 0.74)

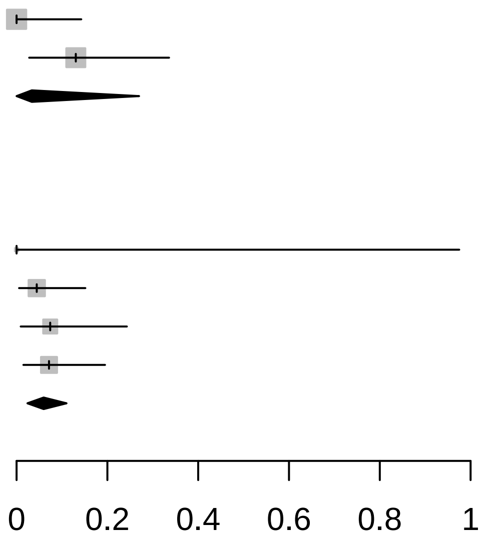

C)

Study	csCancer	Total	Prevalence	95% C.I.
**localization = PZ**				
Brancato 2020	3	17	0.18	[0.04; 0.43]
Rudolph 2020	4	31	0.13	[0.04; 0.30]
**Random effects model**			**0.15**	**[0.06; 0.26]**
Heterogeneity: I^2 = 0%, τ^2 = 0, p = 0.66				
**localization = TZ**				
Byun 2020	23	69	0.33	[0.22; 0.46]
Costa 2020	10	110	0.09	[0.04; 0.16]
Lim 2020	14	95	0.15	[0.08; 0.23]
Rudolph 2020	2	25	0.08	[0.01; 0.26]
Xu 2020	8	17	0.47	[0.23; 0.72]
**Random effects model**			**0.20**	**[0.09; 0.33]**
Heterogeneity: I^2 = 85%, τ^2 = 0.0237, p < 0.01				

Heterogeneity: I^2 = 77%, τ^2 = 0.0177, p < 0.01
Test for subgroup differences: χ^2_1 = 0.42, df = 1 (p = 0.52)

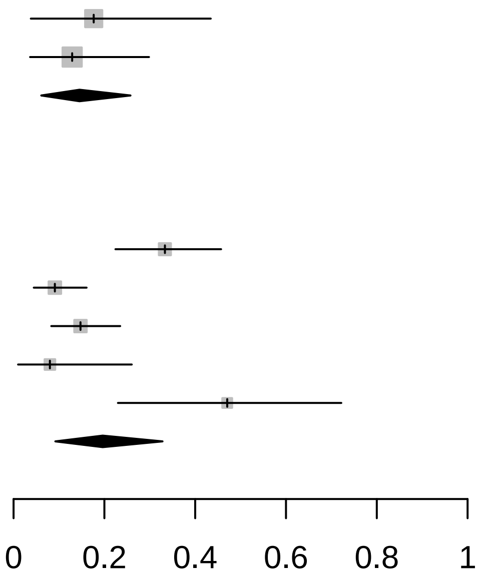

D)

Study	csCancer	Total	Prevalence	95% C.I.
**localization = PZ**				
Brancato 2020	16	38	0.42	[0.26; 0.59]
Rudolph 2020	36	86	0.42	[0.31; 0.53]
**Random effects model**			**0.42**	**[0.33; 0.51]**
Heterogeneity: I^2 = 0%, τ^2 = 0, p < 0.01				
**localization = TZ**				
Brancato 2020	0	2	0.00	[0.00; 0.84]
Byun 2020	17	21	0.81	[0.58; 0.95]
Rudolph 2020	6	15	0.40	[0.16; 0.68]
Xu 2020	4	6	0.67	[0.22; 0.96]
**Random effects model**			**0.50**	**[0.17; 0.82]**
Heterogeneity: I^2 = 78%, τ^2 = 0.0927, p = 0.98				

Heterogeneity: I^2 = 73%, τ^2 = 0.0297, p < 0.01
Test for subgroup differences: χ^2_1 = 0.18, df = 1 (p = 0.67)

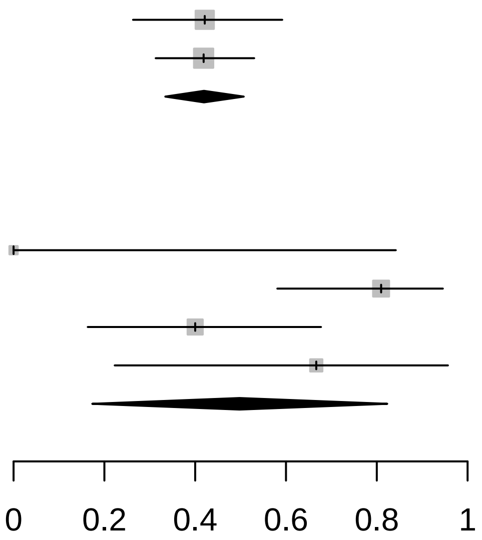

E)

Study	csCancer	Total	Prevalence	95% C.I.
**localization = PZ**				
Brancato 2020	22	26	0.85	[0.65; 0.96]
Rudolph 2020	43	63	0.68	[0.55; 0.79]
**Random effects model**			**0.76**	**[0.58; 0.90]**
Heterogeneity: I^2 = 64%, τ^2 = 0.0123, p < 0.01				
**localization = TZ**				
Brancato 2020	0	2	0.00	[0.00; 0.84]
Byun 2020	29	29	1.00	[0.88; 1.00]
Rudolph 2020	37	60	0.62	[0.48; 0.74]
Xu 2020	10	10	1.00	[0.69; 1.00]
**Random effects model**			**0.79**	**[0.32; 1.00]**
Heterogeneity: I^2 = 94%, τ^2 = 0.2224, p = 0.09				

Heterogeneity: I^2 = 91%, τ^2 = 0.0937, p < 0.01
Test for subgroup differences: χ^2_1 = 0.02, df = 1 (p = 0.89)

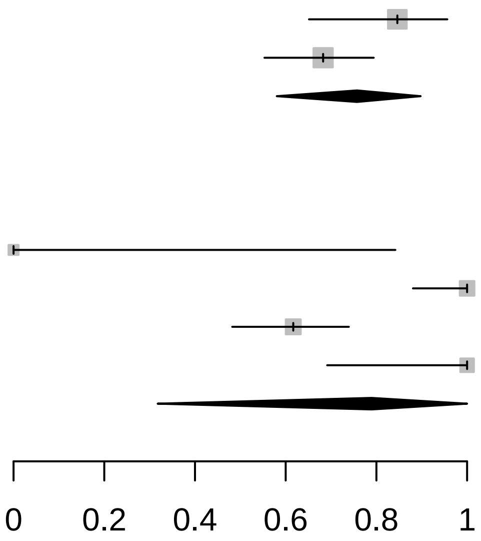

Supplementary figure 5

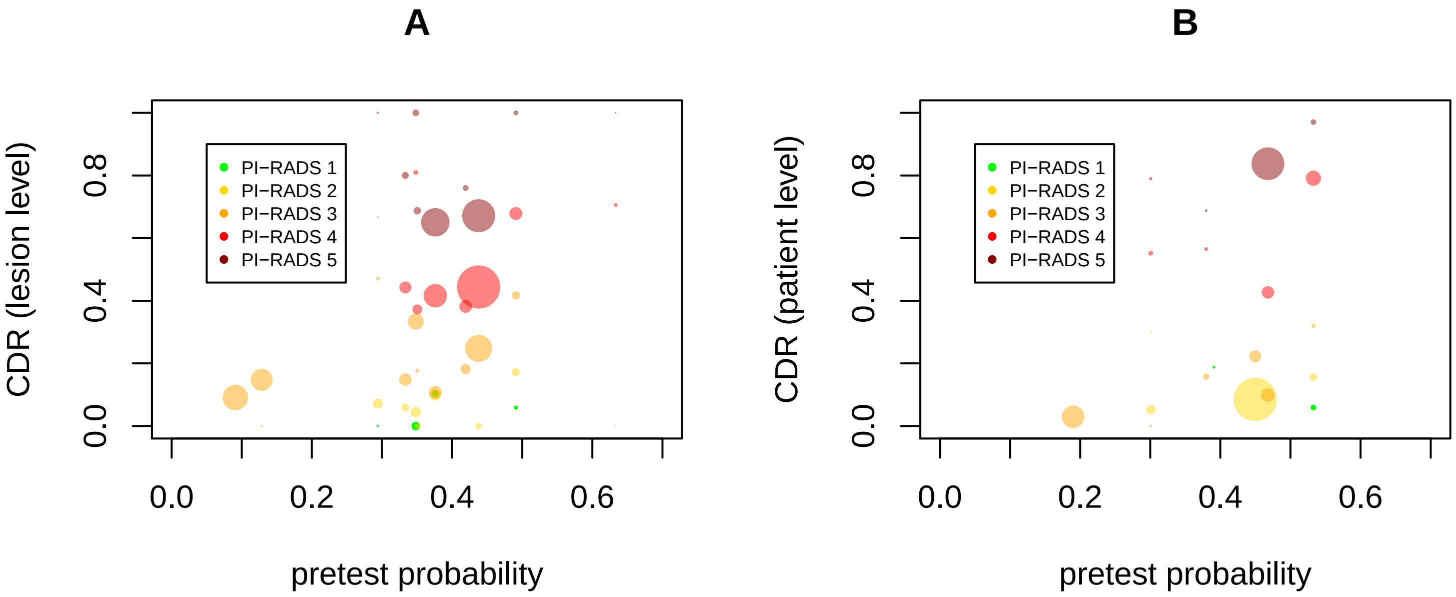

Supplementary figure 6

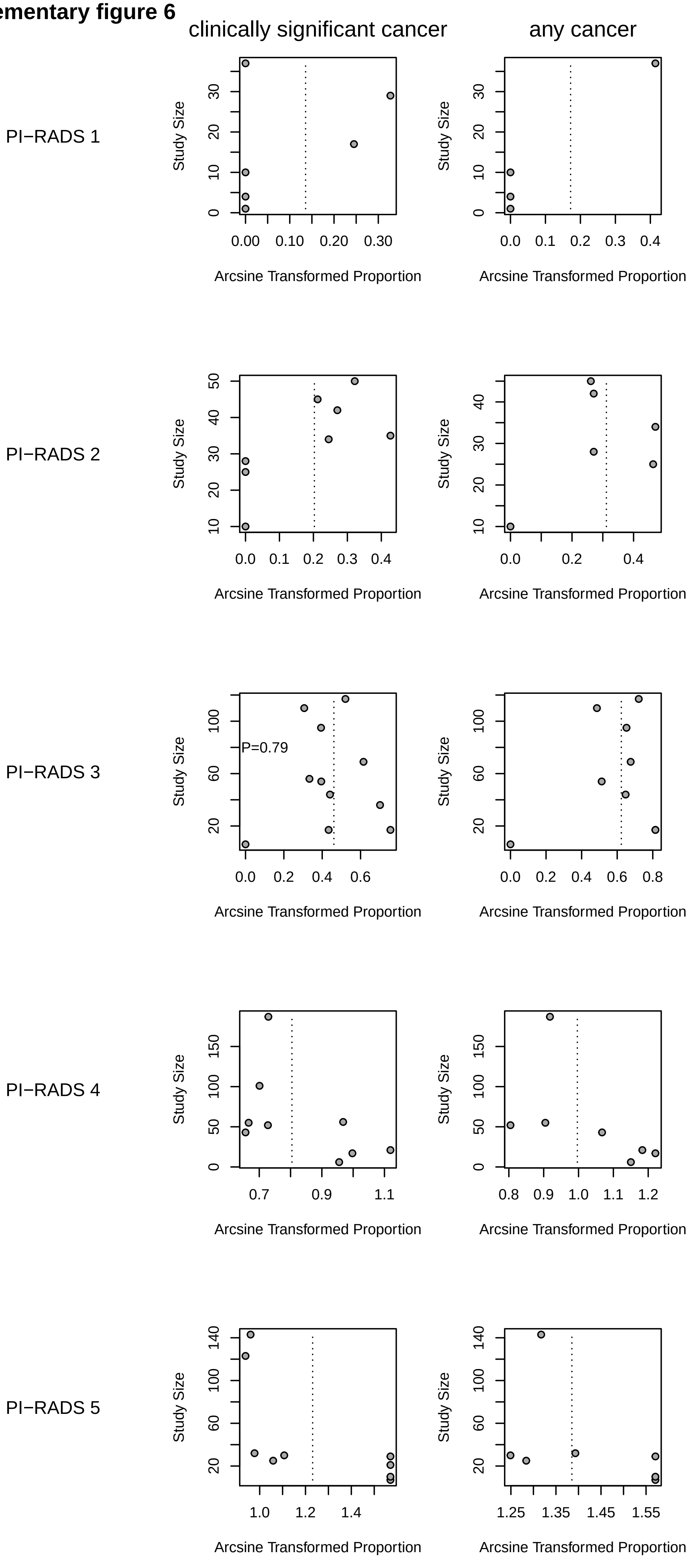

Supplementary figure 7

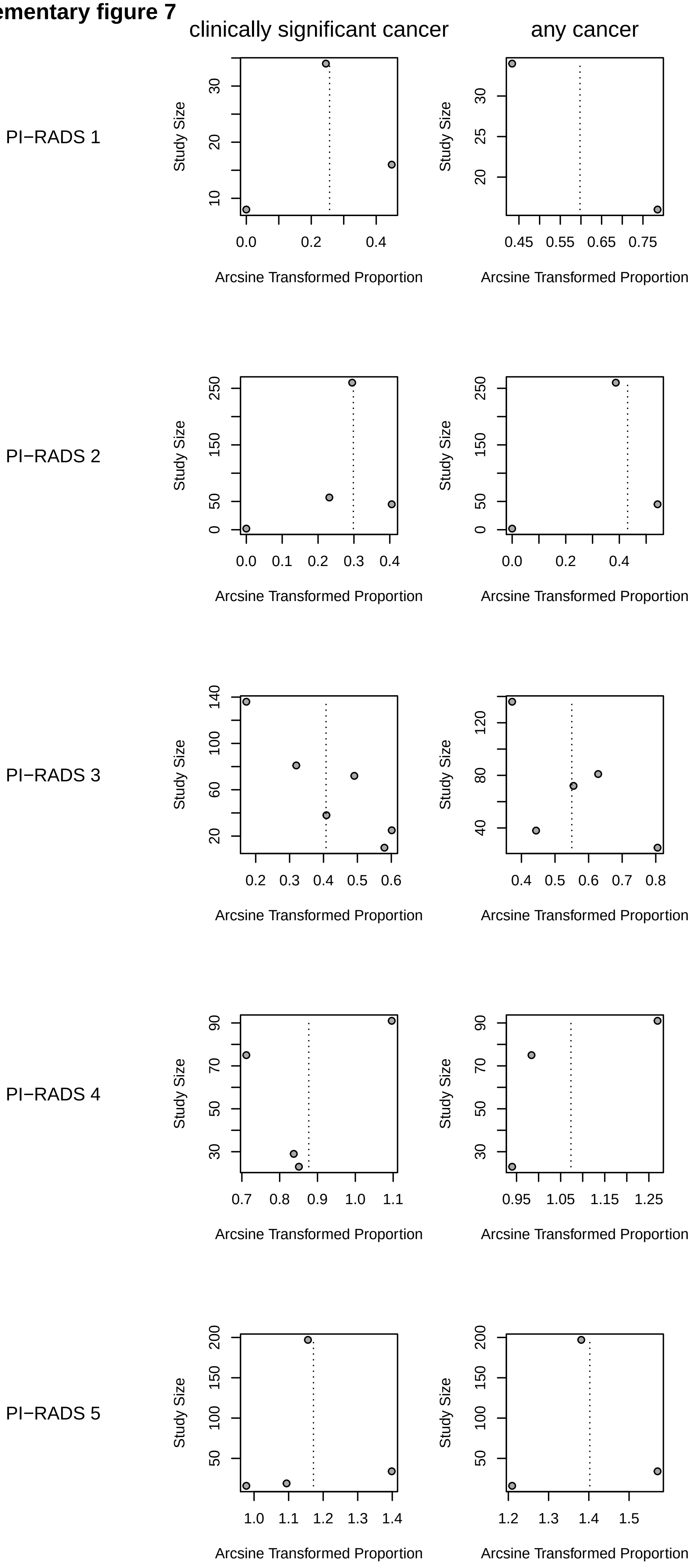

Supplementary Figure Captions

Supplementary figure 1: Forest plots of patient level analysis, cancer detection rates of PI-RADSv2.1 assessment categories for clinically significant cancer as outcome variable. **A**) PI-RADS 1, **B**) PI-RADS 2, **C**) PI-RADS 3, **D**) PI-RADS 4, **E**) PI-RADS 5.

Supplementary figure 2: Forest plots of lesion level analysis, cancer detection rates of PI-RADSv2.1 assessment categories for any cancer as outcome variable. **A**) PI-RADS 1, **B**) PI-RADS 2, **C**) PI-RADS 3, **D**) PI-RADS 4, **E**) PI-RADS 5.

Supplementary figure 3: Forest plots of patient level analysis, cancer detection rates of PI-RADSv2.1 assessment categories for any cancer as outcome variable. **A**) PI-RADS 1, **B**) PI-RADS 2, **C**) PI-RADS 3, **D**) PI-RADS 4, **E**) PI-RADS 5.

Supplementary figure 4: Forest plots of lesion level analysis, cancer detection rates of PI-RADSv2.1 assessment categories for clinically significant cancer as outcome variable, stratified by lesion localization (peripheral zone versus transition zone); given this information is provided. **A**) PI-RADS 1, **B**) PI-RADS 2, **C**) PI-RADS 3, **D**) PI-RADS 4, **E**) PI-RADS 5. From the study of Brancato et al. 7 lesions are excluded from this analysis because they are rated as central zone tumors.

Supplementary figure 5: Dependence of cancer detection rate (clinically significant cancer) on lesion level (**A**) and patient level (**B**) from pretest probability. Size of the data points is proportional to sample size.

Supplementary figure 6: Funnel plots of lesion level analysis. Rows are PI-RADS assessment categories, column 1 is the analysis for clinically significant cancer as outcome variable, column 2 is the analysis for any cancer as outcome variable.

Supplementary figure 7: Funnel plots of patient level analysis. Rows are PI-RADS assessment categories, column 1 is the analysis for clinically significant cancer as outcome variable, column 2 is the analysis for any cancer as outcome variable.

Supplementary Table 1: MRI examinations and case readings

Author (ref. No)	Field strength	One scanner / different scanners	Endorectal coil	Spasmolytic agent	N readers	Years experience prostate MRI*	Slice thickness / GAP T2	FOV T2	Slice thickness / GAP DWI	FOV DWI	high b-Value	Slice thickness / GAP DCE	FOV DCE
Bao [19]	3T	two scanners, same model	no	unclear	2	12	3.5/NR	20x20-25x25	3.5/NR	20x20	2000	3/NR	24x24
Brancato [20]	1.5T	one scanner	yes	unclear	3	10	3/NR	20x20	3/NR	25x25	1400	3.5/NR	20.8x41.7
Byun [21]	3T	different scanners	no	yes	3	>3	3/0	18x18	3/0	18x18	1500	6/0	18x18
Costa [22]	3T	one scanner	yes	unclear	11	20	3/0	18x18	3/0	16x16	2000	NR/NR	NR
Falagario [23]	1.5T and 3T	different scanners	no	unclear	NR	NR	NR/NR	NR	NR/NR	NR	NR	NR/NR	NR
Gorin [24]	NR	NR	NR	unclear	NR	NR	NR/NR	NR	NR/NR	NR	NR	NR/NR	NR
Han [25]	3T	one scanner	no	unclear	2	>5	4/NR	24x24-26x26	4/NR	26x26	1400 (1200 before 2015)	2/NR	26x26 or 36x36
Hosseiny [26]	3T	different scanners	no	yes	4	NR	1.5/NR	21x21	3.6/NR	21x26	1400	3.6/NR	26x26
HosseinyB [27]	3T	different scanners	no	yes	2	15	1.5/NR	21x21	3.6/NR	21x26	1400	3.6/NR	26x26
Hötker [28]	3T	two scanners, same model	mixed	unclear	2	>5	NR/NR	NR	NR/NR	NR	1400	NR/NR	NR
Lim [29]	3T	one scanner	no	unclear	3	7	3/0	22x22	4/0	22x22	1600-2000	4/0	22x22
Rudolph [30]	3T	two scanners, same model	unclear	unclear	3	>5	3/NR	18x18	3/NR	17x17	1400	3/NR	18.6x18.6
Tamada [31]	3T	different scanners	no	yes	3	22	3/0	20x20	3/0	30x30	2000	3.5 or -4/0	25x25 or 35x35
Vilanova [32]	1.5T, NR for second scanner	different scanners	no, outside MRI NR	unclear	2	24	NR/NR	NR	NR/NR	NR	NR	NR/NR	NR
Walker [33]	3T	one scanner	mixed	unclear	1	13	NR/NR	NR	NR/NR	NR	2000	NR/NR	NR
Wang [34]	3T	one scanner	no	unclear	2	10	3/NR	22x22	3/NR	26x26	1000	3/NR	22x22
Xu [35]	3T	one scanner	no	unclear	2	6	3/NR	27x27	3/NR	36x36	1500 or 2000	3/NR	40x40

*most experienced reader, NR: not reported
